# Surgical residents as “second victims” following exposure to medical errors in a tertiary health training facility in Nigeria: a phenomenology study

**DOI:** 10.1186/s13037-023-00370-z

**Published:** 2023-07-18

**Authors:** James Ayokunle Balogun, Adefisayo Ayoade Adekanmbi, Folusho Mubowale Balogun

**Affiliations:** 1 Division of Neurosurgery, Department of Surgery, College of Medicine, University of Ibadan, No. 1, Queen Elizabeth road, University College Hospital Campus, Ibadan, 200001 Nigeria; 2 Department of Neurosurgery, University College Hospital, Ibadan, Nigeria; 3 Institute of Child Health, College of Medicine, University of Ibadan, Ibadan, Nigeria

**Keywords:** Medical errors, Second victim phenomenon, Surgical residents, Health systems, Ibadan

## Abstract

**Introduction:**

The “second victim” phenomenon refers to the distress and other negative consequences that physicians experience when they commit medical error. There has been increasing awareness about this phenomenon and efforts are being made to address it. However, there is dearth of information about it in developing countries. This study explored the experiences of surgical resident doctors of the University College Hospital in Ibadan, Nigeria about the “second victim” phenomenon and the support they had following medical errors.

**Methods:**

This is a phenomenology study in which qualitative data were obtained from interviews with 31 resident doctors across 10 surgical units/departments. Interviews were transcribed verbatim, and data were coded inductively. Data were analyzed using content analysis method. Themes and subthemes were generated using axial coding. The themes were then integrated using selective coding.

**Results:**

There were 31 participants and 10(32.3%) were females. All had witnessed other physicians encountering medical errors while 28(90.3%) had been directly involved in medical errors. Most of the errors were at the inter-operative stage. Prolonged work hours with inadequate sleep were identified as major causes of most medical errors. The feelings following medical errors were all negative and was described as ‘stressful’. Most of the residents got support from their colleagues, mostly contemporaries following medical errors, and many viewed medical errors as a learning point to improve their practice. However, there was a general belief that the systemic support following medical errors was inadequate.

**Conclusion:**

The “second victim” phenomenon was common among the study group with consequent negative effects. Normalizing discussions about medical errors, reduction of work hours and meticulous intraoperative guidance may reduce medical errors and its consequences on the surgical residents. Steps should be taken within the system to address this issue effectively.

## Background

Medical error has continued to be on the front burner of discussions in the medical field, particularly within surgical specialties where adverse events are still regarded as being high [1]. An initial report by the Institute of Medicine (IOM) detailing medical errors as a significant cause of deaths and injuries in the United States [2], validated by the findings of other reports [3–5] had served to provoke discussions around medical errors. Whilst medical errors have been reported across all medical specialties, surgeons have been identified to be more prone to error and with more grievous outcomes [6–9]. Also, the surgical residents are more likely to be involved in medical error compared with residents in the medical subspecialties [10].

Two sets of victims were initially defined in the event of a medical error; the patients and their relatives, described as the first victim, and health care worker involved in the incident, described as the second victims [11]. A third victim, the organization, has also been defined, considering that hospitals function as social organisms and can suffer harm to its reputation and a tendency to lose her inspiration and loyalty of her staff in the event of a medical error [12]. The focus of this paper is on the surgical residents as second victims of a medical error.

The “second victim” phenomenon was first described by Wu in 2000 [13] and relates to the distress felt by a healthcare provider following a medical error [14] or the experience of the health professional involved in a medical error who resultantly becomes emotionally overwhelmed [15]. Scott et al in 2009 put a clear definition to this phenomenon describing “second victims” as healthcare providers who are involved in an unanticipated adverse patient event, in a medical error and/or a patient related injury and become victimized in the sense that the provider is traumatized by the event. Frequently, these individuals feel personally responsible for the patient outcome. Many feel as though they have failed the patient, second guessing their clinical skills and knowledge base” [16]. A more recent consensus definition put the “second victim” as “Any health care worker, directly or indirectly involved in an unanticipated adverse patient event, unintentional healthcare error, or patient injury, and becomes victimized in the sense that also the worker is negatively impacted” [17].

The “second victim” phenomenon is common among health care workers including physicians with a prevalence that can be as high as 30–46% [18, 19]. The phenomenon was reported among about 87% of surgical residents who experienced medical errors and were able to provide details of the event [14]. However, there is limited research on this phenomenon in developing countries and it is important to both appreciate the scope and understand the problem in these settings and in the peculiar context of surgical residency. This will provide baseline data for addressing the problem. Our aim is to investigate the prevalence of the “second victim” phenomenon among residents in surgical specialties and to determine the impact of the adverse event on the second victim, identify the coping strategies employed and explore the possibility of improving on the coping strategies. There is no study to the best of our knowledge in our practice environment that has interrogated this and so, we aim to fill the gap.

## Methods

### Study participants

This study was among resident doctors undergoing training in surgery and surgery sub-specialties. They were at different levels of residency training in the departments of surgery, orthopedics, plastic and reconstructive surgery, neurosurgery, obstetrics and gynecology, ophthalmology and otorhinolaryngology at the University College Hospital, Ibadan.

### Study design, sampling and inclusion criteria

This study used a phenomenology study design to explore the “second victim” phenomenon among surgical residents. The surgical residents were purposively selected if they had completed at least one year of postgraduate training in the surgical and surgical sub-specialties.

### Data collection procedure

Qualitative semi-structured interviews were conducted among trainees in surgical specialties. The semi-structured interviews were open, thus, giving the participants enough flexibility to explore in their responses more deeply and widely. Potential participants were contacted for recruitment into the study mainly by verbal/phone invitations and email exchanges with the study description. The volunteered participants were further informed of the objectives of the study, expectations in terms of publication, and the use of quotations from transcripts, and any risks or benefits that might be incurred by participants in the study. A written informed consent was obtained. All the participants knew the authors as medical doctors prior to the interviews. Also, JB and AA worked in the Surgery department as some of the participants.

Interviews were based on an interview guide adapted from an earlier study on response to catastrophic errors by surgical trainees in Canada [20]. The questions were open ended so that themes could be more fully explored. The interview guide captured the personal and professional demographics, participants’ understanding of medical errors within their specialty, participants’ recall of the circumstances around medical errors they were involved in, physical/psychosocial symptoms experienced, their coping mechanisms and suggestions for desired support. The interviews were conducted in different locations within the hospital depending on the choice of the residents. This was to ensure that the residents were relaxed and not distracted. All the interviews were conducted by AA and were audio recorded. Written informed consent were obtained before each interview and the demographic data including surgical specialty, level of training, age, sex, and marital status were collected. All the residents invited volunteered to take part in the study. Each interview session lasted between nine and 16 min. Data was taken till saturation was reached (no new themes arise during two successive interviews).

### Data analysis

Each audio recording was transcribed verbatim using Express Scribe®. The data was analyzed using content analysis. Each transcript was read closely and coded inductively by a research assistant and FMB. The codes were then reconciled, and axial coding was used to group codes that were related into themes and subthemes as agreed by the two coders. The themes were then integrated through selective coding following critical reviews of the codes within each theme and across the others. Quotes which best represent the themes and subthemes were then selected to illustrate the results.

### Ethical consideration

The ethical approval for the study was obtained from the University of Ibadan/University College Hospital Ethical Review Committee. Participation in the study was voluntary and participants also gave written informed consent. Data from this study were kept strictly confidential. The audiotapes and anonymous transcriptions were kept in a secure location.

## Results

There were 31 residents in this study and 10(32.3%) were females. The participants were residents from different surgical subspecialties as shown in Table 1. The mean age of the participants was 33 ± 2.8 years.

**Table 1:** Sociodemographic characteristics of the surgical residents in the study

Characteristics	Number (n)	Percentage (%)
**Sex**		
Female	10	32.3
Male	21	67.7
**Marital status**		
Single	14	45.2
Married	17	54.8
**Years in residency program**		
2-3years	14	45.2
> 3year	17	54.8
**Specialty**		
Orthopedics	7	22.6
General surgery	6	19.4
Neurosurgery	4	12.9
Cardiothoracic surgery (CTSU)	3	9.7
Ophthalmology	3	9.7
Urology	2	6.5
Plastic surgery	2	6.5
Otorhinolaryngology	2	6.5
Pediatric surgery	1	3.2
Obstetrics and gynecology	1	3.2

The following were the themes and subthemes identified from the transcripts.

- Experiences with medical errors.
  - Types of medical errors experienced/witnessed.
  - Events surrounding the occurrence of medical errors.
  - Effect of medical errors on the residents.
  - Coping strategies following medical errors.
- Impact of medical errors on residents’ practice.
- Support following medical errors.
- Suggestions on how to support residents involved in medical error.

### Experiences with medical errors

All the residents had witnessed other colleagues encountering medical errors in their practice y7yand all but three of them had been directly involved with ME previously.

#### Types of medical errors experienced/witnessed

The medical errors that were common across the specialties were: documentation in the wrong patient’s case file, breach in the maintenance of asepsis and medication errors (including wrong drug, dose and route of administration). Table 2 shows the other medical errors that the residents had been involved in or witnessed.

**Table 2:** Medical errors committed or witnessed by surgical residents in Ibadan

Pre-operative stage errors	Intra-operative stage errors	Post-operative stage errors
Judgment/management	Errors in judgment/management	Delay in wound inspection
Error in drug administration	Technical errors	
	Delay in Surgery	
	Contamination	
	Errors in communication	

#### Events surrounding the occurrence of medical errors

There were some circumstances that promoted the occurrence of medical errors. The commonest was loss of sleep due to prolonged work hours, especially following overnight calls with resultant fatigue as illustrated in the quotes below.

“We are all human beings and prone to errors, when you are sleep deprived as occurs sometimes, you make a few errors here and there.“

“…some people may link it to exhaustion, mental exhaustion, following long period of working.“

The other common circumstance in which medical errors occurred was attempts to adapt equipment for procedures as shown below.

“I was involved in a cataract surgery, I used a heavy loop speculum instead of a light one because that was what was available…that increased the pressure in the eye…”

#### Effect of medical errors on the residents

The medical errors that the residents experienced previously had varying degrees of negative effect on all of them. Negative emotions were used to describe their feelings as shown in theses quotes.

“I felt terrible… I felt really bad.“

“I felt terrible…very stupid, how can I do such thing?“

In an extreme case, a female resident had a shaky voice while recounting her feelings and was visibly uncomfortable discussing it.

There was also self-blaming and expression of loss of self-confidence following ME as shown in the quote below.

“I was not myself, I just felt that if anything should happen to this child, I was the cause of the patient’s death but luckily nothing happened…I gradually gained back my confidence.“

Most of the feelings were also stressful as shown in this quote:

“I was unhappy…unhappiness is a stressful situation…”

“The experience was terrible…it takes a lot of courage to keep practicing with those experiences because it can be demoralizing…”

The way the residents perceived others’ view them after the medical errors also contributed to the already stressful situation:

“Everybody is looking at you as a very bad person, a very irresponsible person…”

However, there were few residents who did not feel so bad following medical errors due to the self-encouragement that they had as seen in these quotes:

“I felt bad, but I did not feel too bad, because I know that we are human, and we all make mistakes.“

“I try as much as possible not to be under tension, or anxious…will I say optimistic on what else we should have done to make sure this patient is stable.“

#### Coping strategies following medical errors

There were different coping strategies employed by the residents following medical errors. One of the commonest was self-encouragement as shown below.

“I guess I felt a little better because I have a mind-set that I can make mistakes if probably I expect myself to be perfect, I will feel a lot worse…”

Some cultures in the units or departments that the residents were also helped them to cope with the effect of medical errors as illustrated in this quote.

“There is a saying in our practice, that the more surgeries you do, and you have not had…those errors, you have not had enough, the more you do, the better you are…”

Interventions to ameliorate the effect of medical errors on the patients also helped the residents to cope:

“Knowing that we were taking care of the problem made me feel better.“

### Impact of medical errors on residents’ practice

The involvement in medical errors made most of the residents to institute some changes in their surgical practice. The commonest change was the resolve to be more careful both in patients’ evaluation and during surgical procedures.

“To be more careful and know my limit… also do a proper pre op evaluation…know when to call your superiors for help.“

“It made me to be more detailed and more careful, in terms of preparing a patient for surgery…”

This made them to institute practices on their own to prevent future occurrences of the errors they were involved in like this resident who decided to develop protocols for his practice.

“…I have decided to have a protocol written for each of the cases I will be handling later in life… the same scrutiny that applies to one patient will be applied to others to minimize each of these errors in the future.“

Some of them also understood better the importance of teamwork in their practice following medical errors as shown in this quote:

“It has affected me a lot, I think I have learnt to listen to even the least person in the team.“

An encounter with medical errors was a career defining moment for one of them, prompting him to come for residency training as shown in this quote:

“I felt bad...that patient was one of the reasons why I had to change my mind to do residency.“

It also helped some of them to overcome the fear that they developed when they get involved with errors in the past due to their resolve not to repeat the same error again as seen in this quote:

“I think it takes away some of the fear and anxiety… being more prepared and a bit less anxious… has helped me move past the errors instead of just dwelling on them...“

### Support following medical errors

Generally, the residents believed the system in which they operate did not have any structured support for them following medical errors as seen below.

“I cannot completely say the institution has a pre-designed support source…there is no clear cut support system.“

However, their colleagues (both senior and contemporary) were their ready source of support many times when they get involved in medical errors. The extent of this support was highly varied across the different specialties. These variations are shown in these quotes:

“In the unit, we do sometimes go over things that happen…when a senior resident tells you that you did the best, it helps to assuage guilt feelings…”

“My colleagues were very supportive, they understood all that happened, they told me the plain truth. They also empathized…I appreciate their role in supporting and correcting me the way they did.“

There were also some residents who mentioned that the senior colleagues did not give them support when they made medical errors and they were left alone just as shown below.

“…If the supervisor is someone that screams on you and is aggressive…most times, the person is left to find a way to solve the problem and ensure that it does not occur again.“

### Suggestions on how to support residents involved in medical errors

There were suggestions from the residents on how they can be supported effectively when they encounter medical errors. One of these is for ME to be seen as systemic errors, which will remove the blame on an individual and promote a holistic view of the situation with effective solution as illustrated in the quote below.

“…The unit needs to see it as a collective error not an individual error…”

There were also suggestions that mentoring can prevent errors as well as reduce its effects on residents as seen in this quote:

“I would imagine there would be a mentor- mentee relationship which will be the best kind of support because it is only a colleague, or a senior colleague that will offer you succor…somebody knowing and accepting that you are a trainee…”

The routine morbidity and mortality meeting was suggested to be an avenue to learn about ME so that errors identified will be learning points, which can be a form of support for those involved in the error and prevent future occurrences.

“The systemic support ideally should be the normal morbidity/mortality meeting… where someone should own up to those errors, you are appropriately guided…”

## Discussion

A lot of attention, understandably so, has been paid to the effect of errors on the patients/relatives who are the primary victims. The impact of medical errors on physicians is being brought to the front burner in the discourse around medical errors [20] because of the level of personal distress associated with self-perceived errors [21]. Some of these experiences have been described as the darkest hours in the careers of professionals involved, that leaves permanent marks on them [16]. A number of findings emerged from this study. Almost all the participants have been “second victims” of medical errors, thus establishing the common occurrence of this phenomenon among the participating residents in our institution. Most of the residents sought and found support from fellow residents who they considered as empathetic and non-judgmental despite not holding back on correcting them regarding the errors. There was very little consideration in seeking help from the consultant staff, mainly so as not to be perceived as incompetent. The residents recommended an organized systemic support for residents who are involved in medical errors. Finally, the experiences formed a fulcrum for positive changes in their practice that were directed at reducing medical errors in their practice.

Our findings of a common occurrence of the “second victim” phenomenon among the participants in our study validates the increasing concern this is generating within the medical cycle due to the confirmed increase in the expression of “second victim” thoughts among physicians [18, 19, 22, 23]. The responses of physicians, including surgical residents to medical errors are varied spanning the cognitive, emotional and behavioral domains [24] and can include guilt, fear, disappointment, sadness, self doubt, frustration and shock [11, 20, 21, 25]. Self-perceived errors could also result in a reduction in quality of life, increased burn out, depression and a decline in empathy [26]. The residents in our study experienced varying types of errors from the pre-operative to the post operative stages but with predominance as expected in the intra-operative stage considering that surgery is both a science and art, with significant emphasis on the technical portions of the training, to produce competent surgeons. The preventable errors and catastrophic errors seem to provoke more intense emotional responses from the residents, understandably because they either involve near misses or there is harm or loss of life. There was really no difference in the ways the residents responded to the medical errors based on their years of training though there seems to be more intense response from those in the middle years of their training. This is likely a reflection of the senior residents being more competent, and more aware of the causes of medical errors from experience and the junior residents not having been exposed to tangible volume of cases. It is important to pay cognizance to the fact that the impact of medical error on residents may be more severe than those of the consultant staff, due to increased vulnerability of residents to shame, which is a deep-seated, painful, and oft-hidden emotion associated with the negative evaluation of the whole self [27].

Scott et al. [16] described six stages in the recovery trajectory of a “second victim” and these include: (i) chaos and accident response, characterized by self reflection focused on verifying exactly what happened, while trying to care for a clinically unstable patient (ii) intrusive reflections stage, which describes a period of isolation and an attempt to re-enact the event and usually associated with feelings of inadequacy and loss of confidence (iii) restoring personal integrity is the stage that the victim seek support from individuals they trust such as colleagues, family and friend, who will understand their experience and the impact of the event, without being judgmental (iv) enduring the inquisition, is the stage at which the individual focus their thoughts on the possible implication of the event on the security of their job (v) obtaining emotional first aid is the phase of attempting to seek emotional support and an awareness of litigation concerns (vi) The ‘moving on’ phase is described as having three paths: dropping out which relates to change of location or exiting the profession or change roles. In the surviving path, the individual continues to function optimally but is hounded by the event, while the thriving path is marked by utilization of the event for something good by either changing practice or being involved in practice change. The intervention offered can be significant in the handling of the medical errors and ultimately in deciding the final pathway to be taken by the residents. Getting the residents to the thriving pathway is particularly important in our practice setting, where there is already significant deficit in surgical manpower and resultant disparity in accessing surgical services. Thus, prevention of impairment or loss of this cohort of trainees acquiring specialized skills becomes a vital tool to breaking the vicious cycle of harm not just to the victims but also to their families, other patients, the hospital and the society at large [28].

Resident physicians have significant emotional challenges when faced with medical mishaps, and have different approaches coping with these difficult experiences [20, 26, 29]. The vulnerability of physicians including residents to emotional and psychological distress from errors has resulted in calls for institutional support [11, 20, 25, 30]. There is also strong advocacy for organizational structure with strategic control of healthcare delivery, and well-embedded incident reporting and adverse events disclosure systems [31]. While all these efforts are veritable tools geared towards addressing the distresses of the residents as “second victims” and can be adapted to each practice environment, we however align with previous suggestions that physicians and residents tend to find colleague residents as a primary coping strategy [23, 32]. Thus, in addition to institutional support, we advocate strengthening the interactions among residents such that it becomes a veritable tool in mitigating the negative effects of medical errors on them. This is particularly important in the field of surgery, which despite continuous modifications remains hierarchical and particularly so in our environment [33]. Interactions can be informal or formal such as resident emotional support groups, and training the residents in skills necessary for debriefing sessions when medical errors occur [32]. The emotional support group can also be designed to be led by residents nominated from among themselves, reflecting the different surgical specialties, who are well respected, and will be trained, thereby becoming the first contact for other residents in the event of medical errors [34]. This will entail providing protected time for the residents that will serve as peer supporters, and that their training is also not negatively impacted. Another challenge of this concept may be the need to mitigate any negative effect on the peer supporters in the process of being a support themselves. Thus, debriefing sessions at fixed intervals with the peer supporters will be helpful and give room to identify those that may want to opt out of the process.

We acknowledge that though our study included a diverse group of residents in surgical specialties, the single center nature of the study and the differing prevailing culture of each practice environment, might limit the generalizability of our findings. The experiences are likely to vary within surgical specialties, which was not accounted for in our study and thus not specialty specific.

## Conclusion

In this study we provided insight into the occurrence of the “second victim” phenomenon within a cohort of residents in surgical subspecialties in our practice environment, documenting the consequences of medical errors on these residents. We also established that most residents got support from their fellow residents following encounters with medical errors, and we proffered how this can be strengthened to mitigate the effects of medical errors on these residents, to achieve thriving residents who will add to the surgical manpower in our environment, ultimately reducing the disparity in accessing safe surgical care.

## Data Availability

The data for this study cannot be made publicly available due to its sensitive nature. However, the data can be obtained from the corresponding author following reasonable request.

## Abbreviations

IOM: Institute of Medicine
